# On Some of the Double Salts of Quinine, and Especially on the Hydrochloro-Sulphate of Quinine

**Published:** 1893-01

**Authors:** 


### ON SOME OF THE DOUBLE SALTS OF QUININE, AND ESPECIALLY ON THE HYDROCHLORO-SULPHATE OF QUININE.

*I. The Chemistry of Hydrochloro-Sulphate of Quinine. By Mons. E. Grimaux.*

In a preceding note I sought to establish that in the basic salts of quinine the acid is united, not to the nitrogen of the quinoleic group, but to the azote of the other group, probably of a piperidinic nature.

It seemed to me, in consequence, that the nitrogen of the quinoleic group might also be made to unite under the influence of an acid, and thus form double salts of quinine, with two different acids; salts which, hitherto, had not been prepared. The trials undertaken on this line permitted me to obtain the hydrochloro-sulphate and the hydriodo-sulphate, as also the corresponding phosphates.

The hydrochloro-sulphate is, in fact, a definite chemical compound and not a mere mixture. When left to itself in dry air, or when its crystaline crust is separated from the mother-waters, and dried on porcelain at 100° of heat, it gives on analysis the same figure as does the total mass.

The hydrochloro-sulphate of quinine is very soluble in water; at 25°, one part of the anhydrous salt dissolves in 1.16 parts of water. It contains 74.2 per cent. of quinine; the medical sulphate ($7H_2O$) contains 74.3 per cent. of quinine.

The hydrated salt fuse, at 125°, resolving into an amber-colored liquid, which forms, on cooling, a gummy mass, Anhydrous, it turns brown in melting, and its fusing point varies indefinitely between 165° and 170°.

If we dissolve the basic sulphate of quinine by means of one-half the quantity of hydrochloric acid, we find that solution can be obtained only by the aid of heat to ebullition, and the use of five parts of water. On cooling, a certain quantity of basic sulphate is separated and the liquor retains hydrochloro-sulphate. The salt, to a single molecule of hydrochloro acid, does not appear to exist here, or, in its solution at least, decomposes into basic sulphate and hydrochloro-sulphate.

Following this report, Mr. E. Grimaux communicated to the Société de Biologié (at the meeting of October 20th, last), the data upon the same subject which we brought together in the last number (44) of the *Tribune Medicale*, page 699.

Professor Laborde took occasion to forward to the same society a report, as follows, of the results which he had obtained in his examination of the physiological action of the new quinine salt.

Mons. E. Grimaux first communicated the following to the French Institute:

*II. Physiological Action of Hydrochloro-Sulphate of Quinine. By Mons. Laborde.*

The tests I have made, said Mons. Laborde, upon animals, of this new salt of quinine, prepared by Mons. Grimaux, have exactly reproduced the symptomatic picture of the physiological and toxic action of quinine.

Characteristic, bilateral agitation of the head, in the guinea-pig; incoördination; motor ataxia; analgesia, localized at first at the point of injection, and afterward becoming generalized; then, at a more advanced stage of the toxic influence, exhilaration and quinic stupor, and, if the dose reaches a toxic total, the phenomena and the processes of terminal asphyxia.

The doses through which these effects were induced, varied, in our experiments, from ten to twenty centigrammes, given in hypodermic injections to guinea-pigs having an average weight of 400 grammes. Even with doses of from two and a half to five centigrammes, we obtained the characteristic phenomena of agitation, incoördination, or quinic intoxication.

But the point in which the new salt is especially distinguished from its simple congeners, notably the sulphate and the hydrochlorate of quinine, lies in the fact of its more rapid absorption, in consequence of which its effects are sensibly more prompt. This is probably due, other qualities being equal, to the much easier and greater solubility of the hydrochloro-sulphate of quinine, as compared with that of the single salts of that base.

From this point of view, the hydrochloro-sulphate of quinine must be regarded as a precious medicament for hypodermic employment, and it is a product, by the way, whose subcutaneous use gives rise to no appreciable, local irritation.

The hydrochloro-sulphate of quinine seems to me to be called to render veritable services to therapeutics.—*La Tribune Médicale*, November 10, 1892.

